# A feasibility pilot study of the effects of neurostimulation on swallowing function in Parkinson’s Disease [version 2; peer review: 1 approved, 3 approved with reservations, 1 not approved]

**DOI:** 10.12688/amrcopenres.13007.2

**Published:** 2022-04-08

**Authors:** Ayodele Sasegbon, Ulrike Hammerbeck, Emilia Michou, Ivy Cheng, Mengqing Zhang, Charlotte James, Shaheen Hamdy

**Affiliations:** 1 Division of Diabetes, Endocrinology and Gastroenterology, University of Manchester, Manchester, Greater Manchester, Stott Lane, Salford M6 8HD, UK; 2 Department of Speech and Language Therapy, University of Patras, Patras, Greece

**Keywords:** Dysphagia, Swallowing, rTMS, PES, Parkinson's

## Abstract

**Introduction:**

Dysphagia often occurs during Parkinson’s disease (PD) and can have severe consequences. Recently, neuromodulatory techniques have been used to treat neurogenic dysphagia. Here we aimed to compare the neurophysiological and swallowing effects of three different types of neurostimulation, 5 Hertz (Hz) repetitive transcranial magnetic stimulation (rTMS), 1 Hz rTMS and pharyngeal electrical stimulation (PES) in patients with PD.

**Method:**

12 PD patients with dysphagia were randomised to receive either 5 Hz rTMS, 1 Hz rTMS, or PES. In a cross-over design, patients were assigned to one intervention and received both real and sham stimulation. Patients received a baseline videofluoroscopic (VFS) assessment of their swallowing, enabling penetration aspiration scores (PAS) to be calculated for: thin fluids, paste, solids and cup drinking. Swallowing timing measurements were also performed on thin fluid swallows only. They then had baseline recordings of motor evoked potentials (MEPs) from both pharyngeal and (as a control) abductor pollicis brevis (APB) cortical areas using single-pulse TMS. Subsequently, the intervention was administered and post interventional TMS recordings were taken at 0 and 30 minutes followed by a repeat VFS within 60 minutes of intervention.

**Results:**

All interventions were well tolerated. Due to lower than expected recruitment, statistical analysis of the data was not undertaken. However, with respect to PAS swallowing timings and MEP amplitudes, there was small but visible difference in the outcomes between active and sham.

**Conclusion:**

PES, 5 Hz rTMS and 1 Hz rTMS are tolerable interventions in PD related dysphagia. Due to small patient numbers no definitive conclusions could be drawn from the data with respect to individual interventions improving swallowing function and comparative effectiveness between interventions. Larger future studies are needed to further explore the efficacy of these neuromodulatory treatments in Parkinson’s Disease associated dysphagia.

## Introduction

Parkinson’s disease (PD) is a common neurodegenerative condition of unclear aetiology wherein there is a build-up of Lewy Bodies within dopaminergic regions of the brain^1^. These Lewy Bodies are primarily composed of the protein alpha synuclein and cause damage to the internal workings of neurones^2,3^. As the disease progresses, there is an increasing burden of pathological protein and an associated decline in neuronal function^1,4,5^. From the point at which a diagnosis of PD is made, patients tend to exhibit an increasing number of symptoms in a predictable manner. As a result, symptomatic scales such as the Hoehn and Yahr scale^6^ are often used to classify PD severity. Epidemiological studies have shown PD is present in up to 4% of people over 55 years of age^7,8^. Although the limb and gait disturbances caused by PD are common and well known^5^, PD is also recognised to cause dysphagia^9^. Dysphagia commonly occurs in patients with PD^10^, with up to 82% of patients developing dysphagia at some point along their illness journey^11^. PD can cause dysphagia directly or indirectly. The direct pathway occurs as a result of Lewy body related damage to swallowing centres within the brain^12^. Conversely, the indirect pathway is due to damage to non-motor brain areas which results in dementia^13^ which in turn causes dysphagia^14^.

At present the management of dysphagia in PD is geared towards compensating for neurological damage with interventions such as dietary modification, altering the consistency of fluids and the use of dopaminergic medications^15,16^. However, a body of evidence exists in support of invasive deep brain stimulation (DBS) for the treatment of PD motor symptoms^17^. DBS delivered to the subthalamic nucleus or globus pallidus is effective at ameliorating motor dysfunction up to 12 months after treatment^17^. There is little data on whether DBS can treat PD dysphagia. Beyond this, neuromodulatory interventions constitute new and emerging developments in the treatment of neurogenic dysphagia. Novel and increasing applied techniques include pharyngeal electrical stimulation (PES) and repetitive transcranial magnetic stimulation (rTMS). PES is a technique whereby a catheter containing two electrodes is inserted transnasally or per-orally into the pharynx. The application of an electric current results in stimulation of sensory afferents supplying the pharynx and increased sensory inflow into brain areas including the sensory and motor cortices^18^. rTMS, by contrast, is a centrally acting as opposed to a peripherally acting technique. It uses a strong electromagnet to pulse magnetic energy at targeted parts of the brain including the swallowing motor cortical areas^19^. High-frequency rTMS (5 Hertz or greater) causes increases in pharyngeal motor cortical neurological excitability^20^ while low frequency (1 Hertz) rTMS causes a suppressive effect^21^.

In PD the nature of Lewy body deposition, neuronal damage and attempted neuroplastic compensation results in cortical areas with decreased activity^22^ and others with increased activity^23^. Within the areas with decreased activity damage can be said to have exceeded compensatory efforts, while in areas with increased activity attempted compensation is ongoing but with unclear effectiveness. Cortical rTMS and PES have been hypothesised to encourage beneficial neuroplastic changes in the brains of patients with neurogenic dysphagia in two distinct ways. Firstly, high frequency (excitatory rTMS) or PES are excitatory and increase neuronal activity over cortical swallowing centres^24^. This increase in activity can either reverse suppressed neuronal activity due to pathological damage, thereby restoring a more normal state of activity^25,26^, or perhaps increase activity in non suppressed areas thereby acting as a trigger to encourage compensation for contralateral damage or restoration of normal activity in areas with disordered neuronal firing. Secondly, low frequency (suppressive rTMS) is thought to block maladaptive neuronal activity in the motor cortex thereby allowing beneficial neuroplastic changes to occur^26^.

Very few non-invasive neurostimulatory studies have been performed in PD with even fewer being performed in the field of dysphagia. Regarding PES, no study has been performed investigating the effects of PES on PD related dysphagia. However, PES has been used in numerous studies as a treatment for post-stroke dysphagia (PSD)^27,28^. A meta-analysis of these studies shows PES is able to improve swallowing performance^29^. Moreover, a single randomised controlled trial utilising high-frequency rTMS in PD dysphagia was performed in 2019 by Khedr *et al*.*^30^*. In that study, rTMS was shown to lead to improvements in a functional dysphagia scale (the Arabic dysphagia handicap score) and pharyngeal transit time for thin fluids and solids^30^. Despite the dearth of rTMS swallowing studies in PD, numerous rTMS studies have been performed in the field of PD limb motor function. While their findings are not directly translatable, they do give an idea of potential swallowing therapeutic effects. These studies have employed both low (1 Hz) and high frequency (5 Hz) cortical targeted rTMS. A meta-analysis of the motor effects of rTMS has shown low-frequency rTMS is able to improve PD limb symptoms^31^. High-frequency rTMS trended towards but did not achieve significance^26^.

## Hypothesis

We hypothesise that rTMS and PES will improve swallowing function in patients with PD associated dysphagia.

## Aims

In patients with dysphagia secondary to PD, we aim to compare the neurophysiological and videofluoroscopic (VFS) swallowing behavioural effects of: Low-frequency rTMS (1Hz), High-frequency rTMS (5Hz) and PES

## Objectives

Our objectives were to generate data establishing proof of concept, feasibility, safety and tolerability.

## Methods

**Figure 1:**
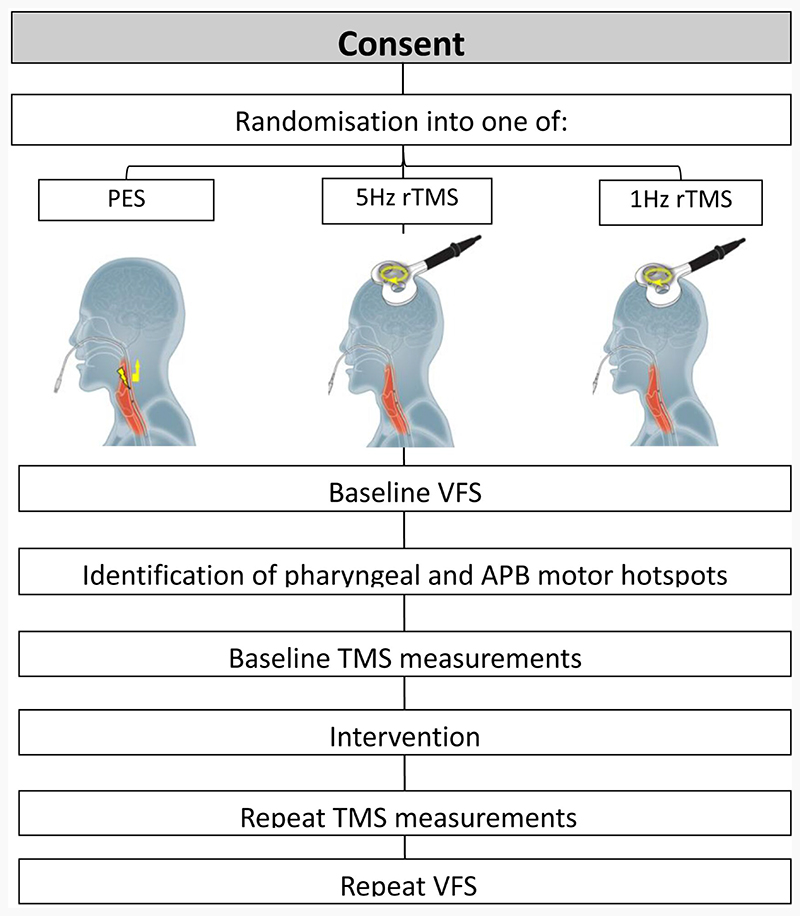
Flowchart illustrating study protocol.

The study was designed as a triple intervention, two-armed crossover, randomised controlled feasibility trial (Figure 1). Although the initial aim was to recruit 66 participants, the COVID-19 pandemic made this unfeasible. For each of the three interventions; 1Hz rTMS, 5Hz rTMS and PES, active stimulation was compared with sham. Over the course of the study, each patient was randomly allocated to one of the three interventions and attended the neuro-motility laboratory on two occasions separated by at least one week. During their initial attendance they received either real or sham stimulation and during their second attendance, the alternative.

The study was assessed and granted ethical approval by the Yorkshire & The Humber - Leeds East Research Ethics Committee (17/YH/0031) and registered on ClinicalTrials.gov (NCT03253354).

### Patient recruitment

Participants were recruited from general neurology clinics, dedicated PD clinics in Salford Royal Hospital (Salford, UK) and PD UK branch meetings.

Inclusion criteria required that patients be diagnosed with PD at least two years prior to the start of the study. Furthermore, patients needed to complain of symptoms of dysphagia, be able to give informed consent and have moderate to severe PD (Hoehn and Yahr Scale II to IV)^6^.

The study exclusion criteria were designed to remove patients: with non-PD causes of dysphagia, with PD mimicking pathologies (multi-system atrophy etc.), lacking capacity to give informed consent and possessing contra-indications for TMS (epilepsy, cardiac pacemakers and metal within the head or neck).

After consenting participants, randomisation to intervention and treatment arms (active or sham) was performed using the statistical website Randola (http://www.rando.la/). Participants then received a screening VFS but only progressed into the study if they had a penetration aspiration score (PAS) of 2 or more, indicating swallowing dysfunction. Patients were blinded (so far as possible) to the intervention they received.

### Symptomatology and activities of daily living

Following randomisation, researchers spoke to participants and completed a Hoehn and Yahr scale^6^ and Schwab and England activities of daily living (ADL) scale^32^.

### Outcome measures

The primary outcome measure for the study was any change between pre- and post-interventional VFS assessed PAS for barium of a ‘thin fluid’ consistency. PAS constitutes an effective means of assessing dysphagia in clinical practice and in research^33^. Cumulative PAS scores were calculated, for primary and secondary PAS outcome measures (see below) and for each thickness or task of barium sulphate swallowed.

Secondary outcome measures included:

1. Change in PAS scores with paste consistency, solid consistency (biscuit covered with barium sulphate) and cup drinking of thin barium sulphate fluid.
2. Swallowing timing measurements during thin fluid swallowing, including oral transit time (OTT), pharyngeal transit time (PTT) and pharyngeal response time (PRT). OTT was defined as the time from bolus propulsion to its passage past the ramus of the mandible into the pharynx. PTT was defined as the time from passage of the bolus into the pharynx to its passage through the upper oesophageal sphincter. PRT was defined as the time from passage of a bolus into the pharynx to elevation of the hyoid.
3. Change in pharyngeal motor evoked potential (PMEP) amplitudes (see study procedures below). Changes in PMEP amplitudes have been shown by previous studies in the field to be correlated with changes in neuronal excitability within the swallowing motor cortex^34^.

### Study protocol

During each session, patients were first taken to the videofluoroscopy (VFS) suite for measurements of their PAS swallowing baseline. Subsequently, they were escorted to the neurophysiology laboratory and seated in a chair. A disposable surgical cap was placed over their heads and secured with medical tape. The location of their cranial vertex was then identified and marked as has been described in previous studies^35^. Abductor pollicis brevis (APB) electrodes and an intraluminal pharyngeal catheter were then positioned. Following this, single-pulse TMS was used to locate pharyngeal motor cortical hotspots bilaterally and the APB motor cortical hotspot on the hemisphere with the lowest pharyngeal resting motor threshold (RMT). RMTs over pharyngeal and APB areas were determined as has been described in previous studies^36^.

Baseline PMEP and APB MEP measurements were obtained by delivering 10 pulses of single-pulse TMS over pharyngeal motor areas bilaterally and the APB area over the ‘dominant’ pharyngeal hemisphere. Following this, either real or sham: 1 Hz rTMS, 5 Hz rTMS or PES was administered. Repeat MEP measurements were then obtained immediately after the intervention and 30 minutes after the intervention. Lastly, participants were taken to the VFS suite for a repeat set of swallowing measurements. A flow chart of the key points of the study protocol can be seen in Figure 1.

### Study procedures

#### Electromyography

Electromyography EMG recordings (allowing measurements of motor evoked potentials) were obtained from the pharynx and the abductor pollicis brevis (APB). Pharyngeal recordings were made using a trans-nasally inserted intraluminal catheter (Gaeltec, Isle of Skye, UK) as described before^37^. APB EMG signals for recording APB MEPs were used as a control and acquired as previously reported^36^.

#### Videofluoroscopy

VFS recordings were obtained with the assistance of trained radiographers. Participants were seated following which the X-ray source and detector were positioned such that lateral views of oropharyngeal structures could be obtained. Images were recorded continuously at 30 frames per second.

Participants were then asked to swallow 10 thin liquid boluses with a volume of 5 ml (barium sulphate w/v ratio of 60%, equivalent to a IDDSI value of 0). Subsequently, they were asked to swallow 3 boluses of a paste consistency (w/v ratio of 40% achieved with ‘Resource Thicken Up Clear’ (Nestle, UK), the equivalent of IDDSI 3) and 3 solid swallows (IDDSI 7). Finally, participants were asked to drink two 50 ml aliquots of thin liquid (IDDSI 0). Barium sulphate (E-Z-Paque, UK) was mixed with water or spread over the surface of solids so as to enable VFS visualisation of boluses. Participants’ VFS PAS data were analysed by a speech therapist blinded to the group assignment. PAS values were obtained for every primary and secondary clearing swallow performed. A primary swallow was defined as the first swallow performed when a bolus was ingested, while secondary or clearing swallows were the subsequent swallow that participants performed to clear any residue. Swallowing timing measurements for thin fluid swallows (OTT, PTT and PTR) were also performed by the same blinded speech and language therapist (IC).

During each study session, participants had baseline and post-interventional VFS recordings. As a safety feature of the study, VFS was stopped if a participant was noted to aspirate more than 50% of bolus volume on 3 consecutive swallows (3 consecutive PAS scores of 8).

#### Single-pulse transcranial magnetic stimulation

Single-pulse TMS was used to elicit motor evoked potentials (MEPs) from pharyngeal and APB motor cortical hotspots. Pulses were delivered using a figure-of-eight electromagnetic coil 7 cm in diameter, with a field strength of 2.2 Tesla, connected to a Magstim Bistim Unit (Magstim, Whitland, UK).

When in use, the coil was held flat against a disposable surgical cap placed over a participant’s head at an angle of 45 degrees. Motor mapping was performed as has been described in several published studies^37,38^. Single-pulse TMS was also used to measure PMEP and APB MEP amplitudes. This was done by delivering 10 pulses of stimulation at 120% of the resting motor threshold (RMT) of the pharyngeal or APB motor areas being studied.

#### Pharyngeal electrical stimulation

PES was delivered using a 3.2mm intraluminal catheter (Gaeltec, Isle of Skye, UK) positioned within the pharynx. The catheter was connected to a signal generator (Digitimer model DS7, Hertfordshire, UK) and a trigger generator (Digitimer Neurology system, Hertfordshire, UK)^39^.

Electrical stimulation was delivered at an intensity determined by the patient’s initial sensory threshold and maximum tolerated sensory threshold. The initial sensory threshold was defined as the intensity of electrical stimulation at which a participant first feels they are being stimulated. The maximum tolerated sensory threshold was defined as the electrical intensity at which patients experienced discomfort. To establish these two thresholds the signal generator current was increased in increments of approximately 0.1mA each second until patients stated that they could feel a sensation in their throat. The intensity at which this occurred was noted and the process repeated twice more. The mean of the three values was then calculated. The maximum tolerated intensity was determined by increasing the electrical intensity further until patients stated that it felt uncomfortable. The intensity of pharyngeal stimulation was set at 75% of the difference between the two values^39^.

Active PES was delivered at a frequency of 5Hz for 10 minutes^39^. For sham PES, the intraluminal catheter was inserted but no electrical stimulation was delivered.

#### Repetitive transcranial magnetic stimulation

RTMS was performed using a Magstim super rapid generator (Magstim, Whitland, UK) connected to a 7cm figure-of-eight coil. High-frequency excitatory rTMS was performed by positioning the coil over the pharyngeal motor cortical area with the lowest RMT (the ‘dominant’ swallowing hemisphere) and delivering 250 pulses at 5 Hz at an intensity of 90% of RMT^28^. Low-frequency suppressive rTMS was also delivered over the pharyngeal motor cortical area, again with the lowest RMT, 600 pulses at a frequency of 1 Hz and an intensity of 120% RMT^24^. Sham rTMS was delivered using the coil tilt technique^35^ where the subject could feel the coil on their scalp and noise of the stimuli, but no energy was delivered to the brain beneath.

### Data analysis

As this study was intended to be a small pilot study exploring the feasibility of using neurostimulatory techniques to induce beneficial changes in swallowing function, only descriptive statistics including means, medians and standard deviations (SD), were used to compare each active treatment (5 Hz rTMS, PES and 1 Hz rTMS) to sham. Hoehn and Yahr and Schwab and England ADL scores were also compared between groups.

#### Penetration aspiration scores

The PAS with the highest numerical value was recorded for each swallow before being added together for each category (thin fluid, paste, solid and cup drinking) to give cumulative penetration aspiration scores^28^. Cumulative scores were converted into percentage differences from individual baseline.

#### Swallowing timing measurments

OTT, PTT and PRT values were obtained from VFS recordings for all interventions for thin fluid swallows before being converted into percentage changes from baseline.

#### Motor evoked potentials

MEP amplitudes were measured in microvolts (μV). MEP latencies were measured as the time in milliseconds (ms) from the point at which a TMS pulse was delivered to the onset of a MEP. MEP amplitude and latency analysis were performed on a desktop computer (DELL, Berkshire UK) using the program Signal (Version 4.0; Cambridge Electronic Design Ltd, Cambridge, UK). The mean of each set of 10 PMEP and APB MEP amplitudes and latencies (at baseline, 0 mins and 30 mins), were obtained before being converted to percentage changes from baseline.

## Results

Patient recruitment commenced in 2019 and was stopped in 2020 during the COVID-19 pandemic due to the mandated cessation of research particularly, as in this case, research that has the potential to be aerosol-generating.

Twelve people with PD (pwPD) were consented and took part in the study (10 males and 2 females with a mean age of 70 (± 8) years) Table 1. Five patients were randomly allocated to the 5 Hz rTMS group (4 male 1 female), 3 to the PES group (3 males) and 4 to the 1 Hz rTMS group (3 males 1 female). Mean ages in the 1 Hz rTMS, 5 Hz rTMS and PES groups were 71 (±8), 67 (±3) and 75 (±12). All neuro-stimulatory procedures were well tolerated, implying clinical feasibility with no adverse effects occurring or being reported by study participants.

The mean Hoehn and Yahr scores in the 1 Hz, 5 Hz rTMS and PES rTMS groups were 2.9 (±0.3), 2.1 (±0.6) and 1.8 (±0.3), respectively. The mean Schwab and England ADL score in the 1 Hz, 5 Hz rTMS and PES groups were 68% (±13), 80% (±12) and 87% (±6).

### Cortical parameters

Seven participants had a dominant right-hemispheric pharyngeal motor area and 5 had a dominant left hemisphere. These remained stable across studies.

The mean pharyngeal RMT over the dominant hemisphere was 77% (±9%) and 69% (±11%) over the non-dominant hemisphere. The mean APB RMT was 43% (±13%).

Using the cranial vertex as a reference point from which to calculate x and y co-ordinates, mean cortical pharyngeal motor areas were located at x = 3.9 cm (± 1.1 cm) and y = 2.6 cm (±1.7 cm) over the right hemisphere and x = -2.5 cm (±3.6 cm) and y = 2.5 cm (±1.7 cm) over the left hemisphere. APB motor areas were located at x = 4.7 (±0.5 cm) and y = 1.3 (±0.9 cm) over the right hemisphere and x = -4.8 cm (±1.4 cm) and y = 1.9 cm (±2.1 cm) over the left hemisphere.

### Penetration aspiration scores

Mean and median cPAS for each group can be seen in Table 2.

#### Thin fluids

**Figure 2:**
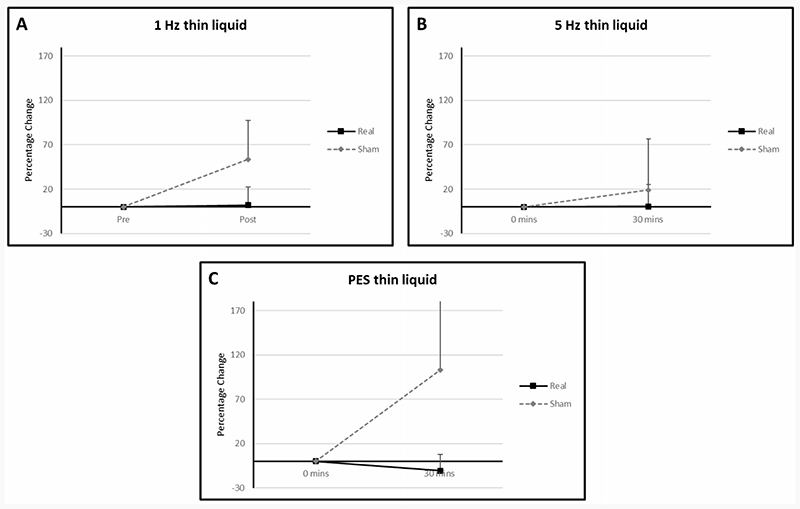
Graphs of percentage differences in PAS for thin fluid in the (**A**) 1 Hz rTMS, (**B**) 5Hz rTMS and (**C**) PES interventional groups. Error bars illustrate standard deviations at each data point.

Mean percentage differences from baseline PAS for thin fluids in the 1 Hz rTMS, 5 Hz rTMS and PES groups were 2.09 (SD: 35.00), 0.49 (SD: 24.50) and -10.53 (SD: 18.23) in the active arms compared with 53.57 (SD: 87.77), 18.97 (SD: 57.83) and 103.25 (SD: 171.36) respectively in the sham arms (Figure 2).

#### Paste

**Figure 3:**
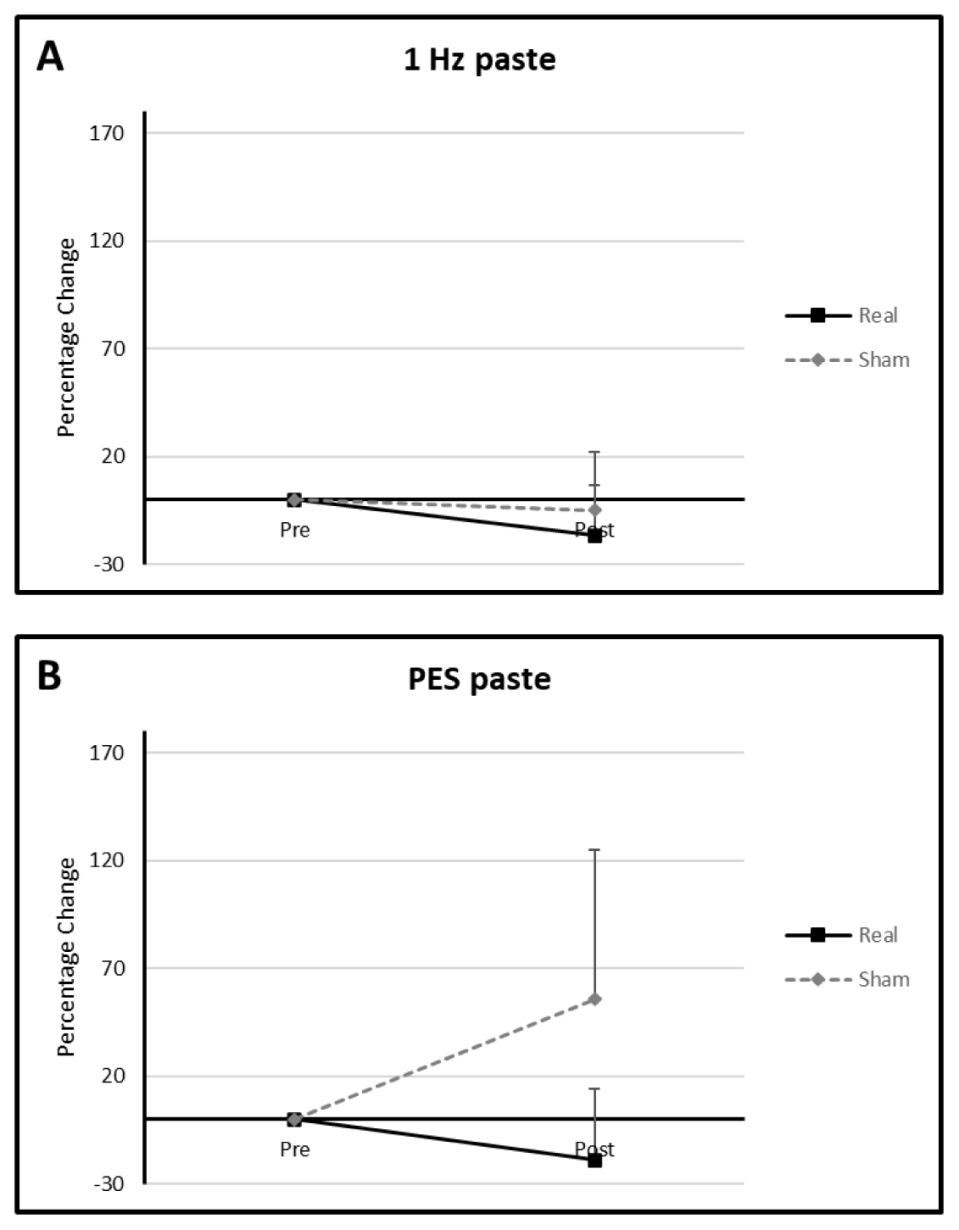
Graphs of percentage differences in PAS for paste consistency in the in the (**A**) 1 Hz rTMS and (**B**) PES interventional groups.

In the active arms mean percentage differences from baseline for the 1 Hz and PES groups were -16.67 (SD: 23.57) and -19.05 (SD: 32.99) compared with -5 (SD: 0) and 55.56 (SD: 69.39) in the sham arms (Figure 3 A+B). The 5 Hz rTMS group could not be analysed as all swallows with paste consistency were <2 (hence normal) for both active and sham arms for all time points.

#### Solid

In the PES group, the mean percentage diference from baseline was -20.0 (SD: 34.64) in the active arm and 122.22 (SD: 107.15) in the sham arm (Figure 3 C). Solid swallows in the 5 Hz and 1 Hz rTMS groups resulted in PAS values of <2 (hence normal) in both active and sham arms. As such no analysis could be performed.

#### Cup drinking (IDDSI 0)

With regards to cup drinking, mean percentage difference in means from baseline in the active 1 Hz rTMS, 5 Hz rTMS and PES groups were 0 (SD: 0), -32.29 (SD: 28.94) and -12.5 (SD: 17.68) respectively compared to -24.44 (SD: 21.43), -4.17 (SD: 54.65) and 0 (SD: 47.14) respectively in the sham arms (Figure 3 D+E).

### Swallow timing results

Raw timing data for thin fluids can be seen in Table 3.

#### Oral transit time

Mean percentage changes in OTT from baseline in the active 1 Hz rTMS, 5 Hz rTMS and PES groups were 16.0 (SD: 42.46), 3.38 (SD: 16.75) and 0.01 (SD: 57.02) and -20.26 (SD: 28.62), 9.02 (SD: 26.70) and 3.71 (SD: 64.56) respectively.

#### Pharyngeal response time

Percentage changes from baseline in the 1 Hz rTMS, 5 Hz rTMS and PES groups were -24.78 (SD: 40.80), 9.29 (SD: 22.26) and -2.83 (SD: 19.58) in the active arms respectively. In the sham arms values were 38.0 (SD: 59.91), 17.44 (SD: 21.84) and 21.86 (SD: 28.46) respectively.

#### Pharyngeal transit time

**Figure 4:**
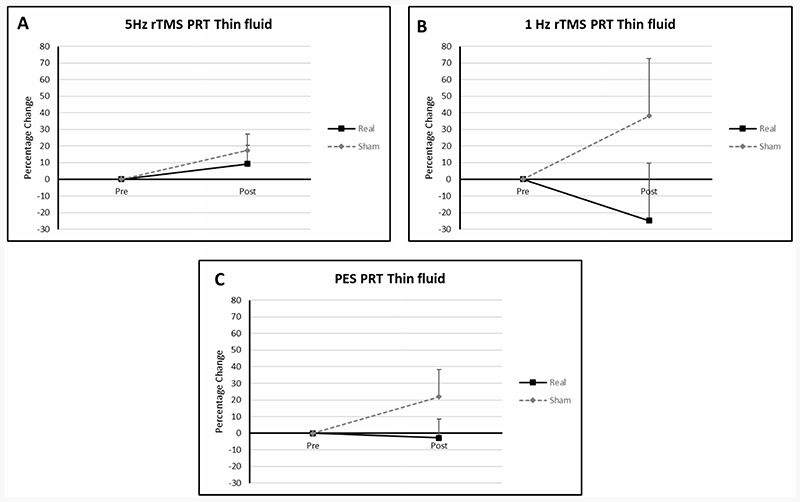
Graphs of percentage differences in PRT in the 1 Hz rTMS, 5 Hz rTMS and PES groups (**A**, **B**, **C**).

Mean PTT percentage changes from baseline in the active arms of the 1 Hz rTMS, 5 Hz rTMS and PES groups were 11.83 (SD: 8.53), 0.66 (SD: 29.79) and 36.72 (SD: 83.61) respectively and 4.53 (SD: 16.52), 24.25 (SD: 35.63) and 30.64 (SD: 34.10) in the sham arms respectively (Figure 4 B).

### Motor evoked potentials

Median values for baseline MEP amplitudes and latencies can be seen in Table 4. Comparing mean percentage changes in amplitudes between ‘dominant’ and ‘non-dominant’ pharyngeal motor hemispheres did not reveal a significant difference for 1 Hz rTMS, 5 Hz rTMS or PES (Paired T-Test: T_5_ =0.99, P =0.37, T_9_ =0.75, P =0.47 and T_5_ =1.76, P =0.14). Hence data were merged to produce a combined hemispheric value as previously reported^24,28^.

#### Amplitudes

##### Pharyngeal

**Figure 5:**
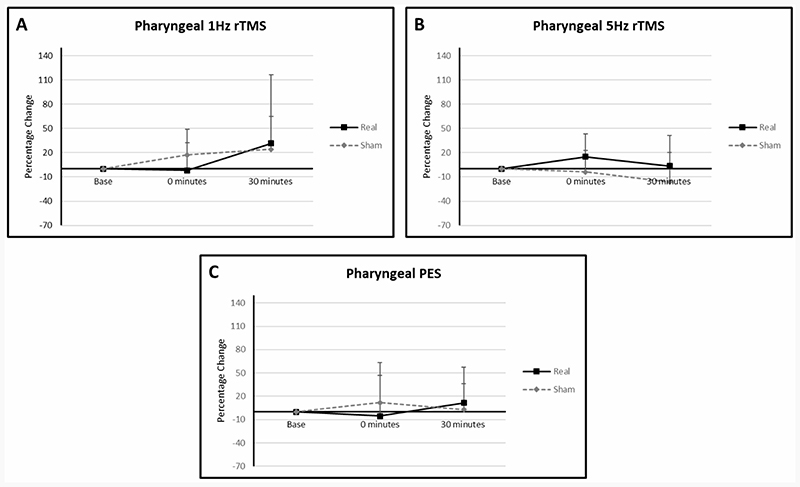
Graphs of percentage differences in PMEP amplitudes in the 1 Hz rTMS (**A**), (**B**) 5Hz rTMS and (**C**) PES interventional groups. Error bars illustrate standard deviations at each data point.

Mean percentage change from baseline PMEP amplitudes in the active arm of the 1 Hz rTMS group were -2.01 (SD: 34.58) at 0 minutes and 31.55 (SD: 85.11) at 30 minutes compared to sham values of 17.30 (SD: 31.55) and 24.34 (SD: 40.70) (Figure 5 A+C). In the 5 Hz rTMS group values in the active arm were 14.98 (SD: 28.43) at 0 minutes and 3.52 (SD: 37.95) at 30 minutes compared to -3.83 (SD: 26.99) and -16.09 (SD: 36.36) in the sham arm. In the active arm of the PES group, values at 0 and 30 minutes were 9.73 (SD: 36.58) and 15.01 (SD: 35.34) compared to 3.93 (SD: 31.92) and -6.63 (SD: 41.17) in the sham arm.

##### APB

Mean percentage changes from baseline for APB MEP amplitudes in the active arm of the 1 Hz rTMS group were -26.49 (SD: 61.25) at 0 minutes and -43.77 (SD: 53.02) at 30 minutes compared to sham values of 35.58 (SD: 33.97) and 32.30 (SD: 35.78) (Figure 5 B+D). In the active arm of the 5 Hz rTMS interventional group percentage changes from baseline were -13.56 (SD: 60.01) at 0 minutes and 25.68 (SD: 57.79) at 30 minutes contrasted with 3.16 (SD: 69.86) and 18.32 (SD: 83.34) in the sham arm. In the PES group, values at 0 and 30 minutes in the active arm were -35.98 (SD: 50.94) at 0 minutes and -49.73 (SD: 68.54) at 30 minutes compared to -13.90 (SD: 61.10) and 71.55 (SD: 82.23).

#### Latencies

##### Pharyngeal

Mean percentage change from baseline PMEP latencies in the active arm of the 1 Hz rTMS group at 0 and 30 minutes were -6.99 (SD: 9.89) and -2.20 (SD: 6.38) compared to sham values of 5.88 (SD: 5.17) and -3.73 (SD: 4.80) (Figure 5 A+C). In the 5 Hz rTMS group, values in the active arm at 0 and 30 minutes were 3.35 (SD: 9.23) and 4.53 (SD: 9.35) compared to -2.73 (SD: 9.13) and 1.07 (SD: 8.60) in the sham arm. In the active arm of the PES group, values at 0 and 30 minutes were 1.49 (SD: 4.05) and -3.41 (SD: 4.88) compared to -0.42 (SD: 4.69) and -2.57 (SD: 1.07) in the sham arm.

##### APB

Mean percentage changes from baseline for APB MEP latencies in the active arm of the 1 Hz rTMS group were -1.63 (SD: 5.62) at 0 minutes and 0.82 (SD: 5.64) at 30 minutes compared to sham values of -1.61 (SD: 2.45) and -5.54 (SD: 5.00) (Figure 5 B+D). In the active arm of the 5 Hz rTMS interventional group percentage changes from baseline were 1.97 (SD: 9.44) at 0 minutes and -2.48 (SD: 4.45) at 30 minutes compared to 5.31 (SD: 7.98) and 1.05 (SD: 4.51) in the sham arm. In the PES group, values at 0 and 30 minutes in the active arm were -1.99 (SD: 7.60) at 0 minutes and 1.87 (SD: 4.18) at 30 minutes compared to -8.75 (SD: 11.02) and 9.09 (SD: 8.90).

## Discussion

Despite the small size of the study, our findings merit further discussion.

### PAS

Interestingly, across all interventions there was a clear graphical separation between active and sham results, with active stimulation consistently having a lower PAS, and hence appearing to be more physiological beneficial, than sham. A potential reason why sham stimulation resulted in higher PAS scores than active stimulation is due to patient fatigue during the course of a study session. It may be that, were more patient data available, statistical analysis and clarity on efficacy may have been possible for one or more interventions. Our findings share some similarities with the results of the only rTMS study performed in PD related dysphagia. In 2019 Khedr *et al* studied 33 patients with PD and found the application of 20 Hz rTMS to the hand motor cortex led to improvements in pharyngeal transit time for thin fluids and solids^30^. However, no significant differences were seen regarding PAS values.

More broadly, a meta-analysis conducted in 2015 by Chou *et al* demonstrated that high-frequency rTMS led to improvements in PD related limb motor dysfunction^40^. However, it should be recognised that the picture regarding the use of high-frequency rTMS to treat PD motor symptoms is a relatively mixed one with another meta-analysis by Shukla *et al* not showing a clear benefit^26^. In the literature, while there are no studies applying 1 Hz rTMS to PD dysphagia, a meta-analysis of the effects of 1 Hz rTMS on motor symptoms in PD showed a significant post interventional improvement^26^. However, similar to the mixed picture for high-frequency rTMS, a recent meta-analysis did not show that 1Hz rTMS can induce motor improvement^40^, therefore, no firm conclusions can be made.

### Swallowing timing

The visual improvements in PRT observed for thin fluids particularly in the 1 Hz rTMS and PES groups are comparable to the improvement in PTT for solids observed by Khedr *et al*. in 2019^30^. These results, imply that both excitatory (PES in this study and 20 Hz rTMS in the Khedr study) and inhibitory (1Hz rTMS) neurostimulation have the potential to affect swallowing physiology and by so doing improve swallowing function.

### MEP

With regards PMEP amplitudes, despite the small number of participants in each interventional group, some minor separation of the trend lines began to emerge between sham and active stimulation for 1 Hz rTMS, 5 Hz rTMS and PES. In more detail, in the 5 Hz rTMS and PES interventional groups, interventions which have been shown to provoke cortical excitation within the swallowing motor system^24,39^, there was the suggestion of greater PMEP amplitudes in the active treatment arms compared to sham. This was also the case following 1 Hz rTMS which is known to cause cortical suppression^41^. Were the groups larger, some significance may have eventually emerged. Despite no previous PD studies having been performed wherein rTMS was delivered to pharyngeal motor cortical swallowing areas (the 2019 Khedr study only stimulated the hand motor area^30^), these findings are tentatively supportive of the multiple studies which show high-frequency rTMS leads to increased PMEP amplitudes^21,24,28^. With regards to 1 Hz rTMS which has been shown to be suppressive when applied over the pharyngeal motor cortex^35^, there was some suggestion that the sham group had greater PMEP amplitudes than the active group. However small numbers make drawing any conclusions from this, premature.

### PD symptom and ADL scores

We did note that the H&Y score was higher in the 1Hz rTMS interventional group than the other intervention arms. Despite participants being allocated at random, this indicates participants in the 1 Hz rTMS group had slightly more severe PD symptoms than those in the other groups. By contrast, there were no differences in Schwab and England ADL scores across any of the intervention arms. The significance of the Hoehn and Yahr differences is unclear given similar ADL performances between groups which implies that participants in the 1Hz rTMS group were still as fit and able as participants in the other interventional groups.

### Limitations

Our study has several limitations. First, the number of patients that were able to be recruited was small. Patient recruitment was negatively impacted by several issues many of which were logistical and not in the control of the research team. Some examples include: there was some anecdotal evidence that emerged during the study which suggested patients with moderate PD were not as troubled by their relatively mild dysphagia as they were by their limb motor symptoms. This may explain why relatively few patients reached out to the research team regarding study participation. Conversely, patients with severe dysphagia were often too frail to be studied in a laboratory setting.

Another limitation was the onset of COVID-19 pandemic and research restrictions that were put in place to prevent the spread of the virus. Swallowing research, especially research involving pharyngeal intubation, is potentially aerosol generating meaning patient recruitment was stopped more than 6 months prior to the planned end date. This reduction in recruitment lead to reduced power and hence contributed to difficulty in drawing definitive conclusions from the study

MEP recordings were only made up to 30 minutes post-stimulation. This was done to reduce the time patients had to be present in the laboratory thereby making the experience more tolerable and reducing dropout. However, most healthy participant neurophysiological studies which measure MEP amplitudes record for up to an hour post stimulation^24,42^. Furthermore, in these studies, maximal separation between interventional groups tends to occur at times between 30 and 60 minutes^37,42^. Therefore, in only making recordings up to 30 minutes post intervention, any delayed effects of neurostimulation might be missed.

Lastly, PAS was used to assess swallowing function within our study. However, while it remains a commonly used and validated method of swallowing assessment, in clinical practice and in research, it is not a perfect assessment of swallowing function^33^. One of its limitations is its inability to quantify the amount of each bolus that is aspirated^33^.

## Conclusion

In conclusion, the use of neurostimulation in patients with PD dysphagia is well tolerated and might lead to some improvements in swallowing function, however suboptimal recruitment precludes more definitive conclusions. Larger studies will be needed to further answer the important question of does neuromodulation improve swallowing in PD associated dysphagia, in this understudied area of medicine.

## Supplementary Material

Full Text XML

## Figures and Tables

**Table 1:** Demographic data for study participants.

Participants	Intervention	Sex	Age	Time since PD diagnosis	Hohen and Yarr	Schwab and England
1	1Hz	M	78	4	2.5	0.7
2	5Hz	M	63	3	2	0.8
3	5Hz	F	70	3	1.5	0.8
4	PES	M	62	5	1.5	0.8
5	1Hz	M	60	8	3	0.8
6	1Hz	M	70	5	3	0.7
7	1Hz	F	76	6	3	0.5
8	5Hz	M	66	2	2	0.9
9	PES	M	83	3	2	0.9
10	5Hz	M	67	7	2	0.9
11	PES	M	81	6	2	0.9
12	5Hz	M	69	5	3	0.6

**Table 2:** cPAs data for each interventional group.

			Mean		Median	
			Pre	Post	Pre	Post
**1 HZ**	**Active**	**Thin**	18.8 ± 5.9	18.0 ± 2.1	21.5	15.0
		**Paste**	4.5 ± 1.7	3.5 ± 0.0	4.5	3.5
		**Solid**	3.0 ± 0.0	3.0 ± 0.0	3.0	3.0
		**Cup drinking**	5.0 ± 3.5	3.0 ± 0.0	4.0	3.0
	**Sham**	**Thin**	14.0 ± 8.4	16.0 ± 1.1	15.5	14.5
		**Paste**	3.8 ± 1.0	3.5 ± 0.4	3.5	3.0
		**Solid**	3.3 ± 0.5	3.3 ± 0.2	3.0	3.0
		**Cup drinking**	6.0 ± 3.7	3.0 ± 0.0	5.5	3.0
**5 HZ**	**Active**	**Thin**	12.3 ± 3.3	13.0 ± 5.6	11.0	11.0
		**Paste**	3.0 ± 0.0	3.0 ± 0.0	3.0	3.0
		**Solid**	3.0 ± 0.0	3.0 ± 0.0	3.0	3.0
		**Cup drinking**	6.8 ± 3.0	3.6 ± 1.3	7.0	3.0
	**Sham**	**Thin**	12.8 ± 4.7	13.6 ± 6.1	11.0	10.0
		**Paste**	3.0 ± 0.0	3.0 ± 0.0	3.0	3.0
		**Solid**	3.0 ± 0.0	3.0 ± 0.0	3.0	3.0
		**Cup drinking**	4.6 ± 1.9	4.3 ± 2.2	4.0	4.0
**PES**	**Active**	**Thin**	12.3 ± 5.9	10.3 ±2.5	10.0	10.0
		**Paste**	6.7 ± 6.4	4.0 ± 1.7	3.0	3.0
		**Solid**	5.3 ± 4.0	3.3 ± 0.6	3.0	3.0
		**Cup drinking**	4.0 ± 0.0	3.5 ± 0.7	4.0	3.5
	**Sham**	**Thin**	10.0 ± 7.0	12.3 ± 4.0	13.0	13.0
		**Paste**	3.0 ± 0.0	4.7 ± 2.1	3.0	4.0
		**Solid**	3.0 ± 0.0	6.7 ± 3.2	3.0	8.0
		**Cup drinking**	4.5 ± 2.1	5.0 ± 4.2	4.5	5.0

**Table 3:** Swallowing timing data.

		Active			
		Mean (ms)		Median (ms)	
		Pre	Post	Pre	Post
**1 Hz**	**OTT**	362 ± 193	470 ± 416	301	250
	**PRT**	681 ± 646	395 ± 274	423	250
	**PTT**	447 ± 168	501 ± 198	372	392
**5 Hz**	**OTT**	344 ± 133	343 ± 193	365	380
	**PRT**	180 ± 325	216 ± 212	193	234
	**PTT**	411 ± 117	387 ± 51	456	407
**PES**	**OTT**	298 ± 73	310 ± 188	276	417
	**PRT**	547 ± 443	496 ± 334	303	362
	**PTT**	464 ± 192	578 ± 257	360	570
		**Sham**			
		**Mean (ms)**		**Median (ms)**	
		**Pre**	**Post**	**Pre**	**Post**
**1 Hz**	**OTT**	470 ± 241	344 ± 121	396	310
	**PRT**	519 ± 512	922 ± 1182	298	335
	**PTT**	510 ± 161	548 ± 256	421	434
**5 Hz**	**OTT**	374 ± 82	419 ± 183	372	395
	**PRT**	268 ± 160	307 ± 161	198	288
	**PTT**	351 ± 83	414 ± 38	310	400
**PES**	**OTT**	419 ± 128	389 ± 196	360	350
	**PRT**	480 ± 252	560 ± 219	389	598
	**PTT**	326 ± 80	439 ± 215	288	360

**Table 4:** Median (+/- interquartile range) cortical pharyngeal and cortical APB MEP amplitudes in microvolts (μV) and latencies in milliseconds (ms).

	5 Hz rTMS		PES		1 Hz rTMS	
	Baseline	30mins	Baseline	30mins	Baseline	30mins
***MEP amplitudes (μV)***						
**Cortical pharyngeal**	90.9 ± 27.4	103.1 ± 7.7	62.2 ± 23.7	67.1 ± 19.3	253.5 ± 71.7	169.6 ± 63.6
**Cortical APB**	47.8 ± 66.0	41.3 ± 114.9	573.8 ± 10.8	252.6 ± 92.2	2431.5 ± 1260.5	251.6 ± 548.0
***MEP latencies (ms)***						
**Cortical pharyngeal**	7.9 ± 1.1	8.6 ± 0.7	8.9 ± 0.3	8.6 ± 0.2	9.7 ± 0.5	10.1 ± 0.6
**Cortical APB**	25.3 ± 2.1	24.4 ± 1.5	23.1 ± 1.1	23.2 ± 1.0	23.7 ± 0.4	24.4 ± 0.7

## Data Availability

Figshare. Parkinsons study data AOS.xlsx. DOI: https://doi.org/10.48420/14958540.v1^43^ This project contains the following data: - Data from a feasibility pilot study of the effects of neurostimulation on dysphagia recovery in Parkinson’s Disease Data are available under the terms of the Creative Commons Zero “No rights reserved” data waiver (CC BY 4.0 Public domain dedication). Figshare. Study Protocol: Exploring Novel Neurostimulation Based Therapies for Swallowing Impairments in Parkinson’s Disease. DOI: https://doi.org/10.48420/14995077.v1^44^ Figshare. CONSORT checklist for study “A feasibility pilot randomised controlled study of the effects of neurostimulation on dysphagia recovery in Parkinson’s Disease” DOI: https://doi.org/10.48420/15082662.v1^45^ Figshare. CONSORT flowchart for the study “A feasibility pilot randomised controlled study of the effects of neurostimulation on dysphagia recovery in Parkinson’s Disease”. DOI: https://doi.org/10.48420/15082674.v2^46^

## Revised Amendments from Version 1

The paper has been adjusted in line with the suggestions from the two peer reviewers. Changes include:

- The study title has been changed. It now reads “A feasibility pilot study of the effects of neurostimulation on swallowing function in Parkinson’s Disease”
- Further justification in the introduction for using neuromodulation to address dysphagia in Parkinson’s Disease (PD)
- Stand-alone aim and hypothesis sections as opposed to these sections being integrated into the text at the end of the introduction
- Changes to the layout of the methods section. These include moving the protocol section, so it appears earlier in the section and minor changes to abbreviations.
- The results section now incorporates text referring to tolerability
- The limitations section of the paper has been expanded to include information stating the penetration aspiration score (PAS) is not without its drawbacks when it comes to assessing swallowing function.

**Any further responses from the reviewers can be found at the end of the article**
